# John Knowsley Thornton

**Published:** 1904-03

**Authors:** 


### OBITUARY.

John Knowsley Thornton, M. B., C. M. Edin., died at his residence, Hildersham Hall, Cambridge, Eng., January 3, 1904, aged 58 years. Mr. Thornton was at one time one of the leading abdominal surgeons in London and performed distinguished service for several years at the Samaritan Fever Hospital. He was also authority on abdominal surgery in which special line of work he developed rare skill, great acumen and masterful results. He began as an assistant to Sir Spencer Wells and finally achieved the highest fame as a bold, conscientious and careful operator. His commanding presence at the operating table dominated assistants, nurses, and visitors, and one did not need to be long in his presence to recognise his manly and professional worth.

He fell ill in 1896, suffering repeated attacks of influenza, complicated by gout. In 1898 he retired from practice and, until his death, lived at Hildersham Hall, near Cambridge. He sought, through work in his garden and farmyard, to restore his lost vigor, a struggle which vainly continued through much suffering until peripheral neuritis finally exhausted his strength. He was married twice: first, to Miss Patterson, by whom he had a son and two daughters, the former holding an appointment in South Africa; his second wife was the Hon. Mary Windsor Clive, by whom he had a daughter; they both survive him.

Mr. Thornton was an Honorary Fellow of the American Association of Obstetricians and Gynecologists.

